# Supplementary material for: Genotypic analysis of the female BPH/5 mouse, a model of superimposed preeclampsia
Source: PLoS One. 2021 Jul 16;16(7):e0253453. doi: 10.1371/journal.pone.0253453 (PMC8284809; doi:10.1371/journal.pone.0253453)
Supplement: S7 Table — (DOCX) [file pone.0253453.s007.docx]

[10.6084/m9.figshare.14888202](https://doi.org/10.6084/m9.figshare.14888202)
